# Environmental Calcium Initiates a Feed-Forward Signaling Circuit That Regulates Biofilm Formation and Rugosity in Vibrio vulnificus

**DOI:** 10.1128/mBio.01377-18

**Published:** 2018-08-28

**Authors:** Daniel M. Chodur, Patrick Coulter, Jacob Isaacs, Meng Pu, Nico Fernandez, Chris M. Waters, Dean A. Rowe-Magnus

**Affiliations:** a Department of Biology, Indiana University—Bloomington, Bloomington, Indiana, USA; b Department of Molecular and Cellular Biochemistry, Indiana University—Bloomington, Bloomington, Indiana, USA; c Department of Microbiology and Molecular Genetics, Michigan State University, East Lansing, Michigan, USA; University of Washington

**Keywords:** PAPS, *Vibrio*, biofilms, c-di-GMP, extracellular signaling, metabolic regulation, second messenger

## Abstract

Poor clinical outcomes (disfigurement, amputation, and death) and significant economic losses in the aquaculture industry can be attributed to the potent opportunistic human pathogen Vibrio vulnificus. V. vulnificus, as well as the bivalves (oysters) it naturally colonizes, is indigenous to estuaries and human-inhabited coastal regions and must endure constantly changing environmental conditions as freshwater and seawater enter, mix, and exit the water column. Elevated cellular c-di-GMP levels trigger biofilm formation, but relatively little is known regarding the environmental signals that initiate this response. Here, we show that calcium is a primary environmental signal that specifically increases intracellular c-di-GMP concentrations, which in turn triggers expression of the *brp* extracellular polysaccharide that enhances biofilm formation. A transposon screen for the loss of calcium-induced *P*_*brpA*_ expression revealed CysD, an enzyme in the sulfate assimilation pathway. Targeted disruption of the pathway indicated that the production of a specific metabolic intermediate, 3′-phosphoadenosine 5′-phosphosulfate (PAPS), was required for calcium-induced *P*_*brpA*_ expression and that PAPS was separately required for development of the physiologically distinct rugose phenotype. Thus, PAPS behaves as a second messenger in V. vulnificus. Moreover, c-di-GMP and BrpT (the activator of *brp* expression) acted in concert to bias expression of the sulfate assimilation pathway toward PAPS and c-di-GMP accumulation, establishing a feed-forward regulatory loop to boost *brp* expression. Thus, this signaling network links extracellular calcium and sulfur availability to the intracellular second messengers PAPS and c-di-GMP in the regulation of V. vulnificus biofilm formation and rugosity, survival phenotypes underpinning its evolution as a resilient environmental organism.

## Abstract (precis)

The second messenger c-di-GMP is a key regulator of bacterial physiology. The V. vulnificus genome encodes nearly 100 proteins predicted to make, break, and bind c-di-GMP. However, relatively little is known regarding the environmental signals that regulate c-di-GMP levels and biofilm formation in V. vulnificus. Here, we identify calcium as a primary environmental signal that specifically increases intracellular c-di-GMP concentrations, which in turn triggers *brp*-mediated biofilm formation. We show that PAPS, a metabolic intermediate of the sulfate assimilation pathway, acts as a second messenger linking environmental calcium and sulfur source availability to the production of another intracellular second messenger (c-di-GMP) to regulate biofilm and rugose colony formation, developmental pathways that are associated with environmental persistence and efficient bivalve colonization by this potent human pathogen.

## INTRODUCTION

Bacteria can exist as free-living, planktonic cells or in surface-attached, multicellular biofilm communities that are composed of bacteria encased in a matrix of polysaccharides, DNA, and proteins (1). Biofilms are more resistant than their planktonic counterparts to environmental stressors such as antibiotic exposure, oxidative agents, ionic stress, and desiccation. The process of biofilm formation is tightly controlled at multiple stages, from approaching and probing a surface, to irreversible attachment and maturation, to biofilm dispersal (2). The second messenger c-di-GMP is a key biofilm regulatory molecule (3), and bacteria can sense diverse environmental signals and modulate their intracellular c-di-GMP levels accordingly. It is synthesized by diguanylate cyclases (DGCs) that bear a GGDEF domain and is degraded by phosphodiesterases (PDEs) that harbor EAL or HD-GYP domains (4). The GGDEF and EAL/HD-GYP domains of DGC and PDE are often associated with sensor domains that regulate their function in response to external cues such as oxygen level, light, nitric oxide, temperature, nutrient limitation, and surface contact (5). Additionally, DGC and PDE activity can be regulated at the level of gene expression. The downstream effects of c-di-GMP are mediated by effectors (proteins or nucleic acids such as riboswitches) that bind the molecule and elicit a change in gene expression or protein function (6).

In Vibrio vulnificus, elevated levels of intracellular c-di-GMP trigger biofilm formation by inducing expression of the *brp*-encoded exopolysaccharide (EPS) (7, 8). Excessive production of EPS and matrix proteins promotes development of the rugose phenotype, which is characterized by a distinct wrinkly colony morphology, and increased biofilm formation and stress resistance (9–11). *brp* expression is also dependent on the regulators BrpR and BrpT (7, 12). BrpR shares homology with VpsR of Vibrio cholerae, which has been shown to bind c-di-GMP (13, 14). BrpT binds directly to promoter regions upstream of *brpA* and *brpH*, but unlike its V. cholerae homologue VpsT, it does not depend on first binding c-di-GMP to do so (12, 15). However, the expression of *brpT* is regulated by BrpR (12). It was also recently shown that c-di-GMP, via BrpT, regulates the expression of the *cabABC* operon (16), which encodes a system for the secretion of a calcium binding matrix protein CabA that is required for biofilm and rugose colony formation (17).

The V. vulnificus genome encodes nearly 100 proteins predicted to synthesize, degrade, and bind c-di-GMP (18, 19), but relatively little is known regarding the environmental signals that regulate c-di-GMP levels and biofilm formation in response to changing environmental conditions. Vibrio vulnificus and the bivalves (oysters) it colonizes are autochthonous to estuary ecosystems (20, 21). These partially enclosed bodies of water are in constant flux due to the varying flows of freshwater (rainfall and snowmelt) and seawater (changing tides) that enter, mix with, and exit the water column (22). The salinity can range from 5 to 30 ppt, vary between estuaries, and change daily depending on the tides, weather, and anthropogenic factors. It was previously reported that calcium increased phase variation and biofilm formation in V. vulnificus (23, 24), but the mechanism remained nebulous. Here, we identify calcium as a primary environmental signal that specifically increases intracellular c-di-GMP concentrations, which in turn triggers subsequent *brp*-mediated biofilm formation. A screen for transposon (Tn) mutants that failed to respond to increased calcium revealed that sulfate assimilation is genetically linked to *brp* expression. We show that a specific intermediate of the sulfate assimilation pathway, 3′-phosphoadenosine 5′-phosphosulfate (PAPS), is required for calcium-induced *brp* expression. Moreover, PAPS production was also separately required for development of the physiologically distinct rugose phenotype. These results support the notion that PAPS is an intracellular second messenger linking environmental calcium and sulfur source availability to the production of another intracellular second messenger (c-di-GMP) to regulate the physiological responses of biofilm formation and rugosity, phenotypes that underpin the evolution of V. vulnificus as a successful environmental organism.

## RESULTS

### Environmental calcium regulates *brp* expression by moderating the intracellular c-di-GMP level.

**FIG 1:**
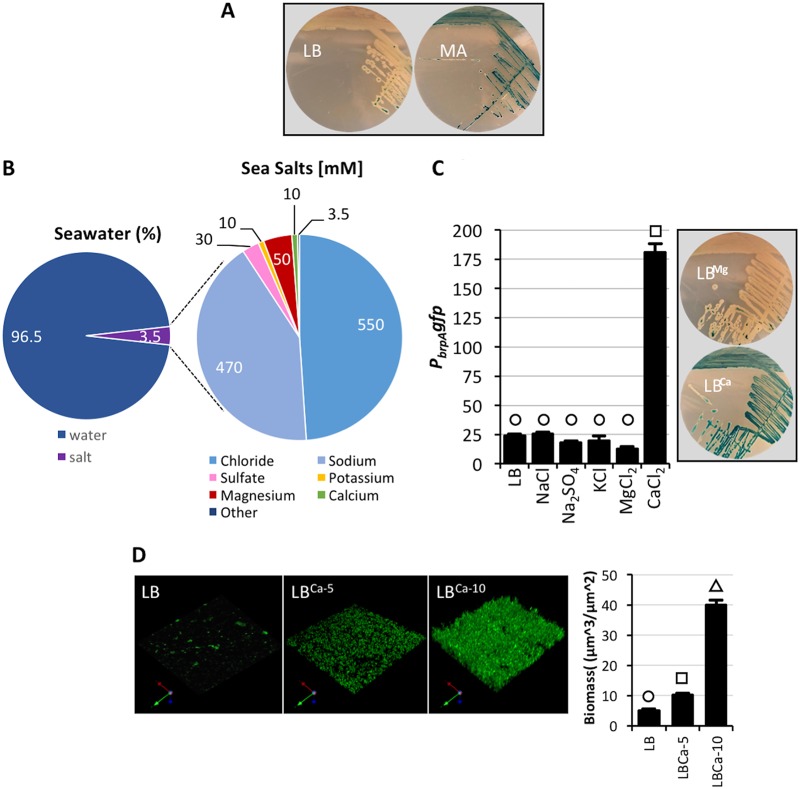
Environmental calcium specifically regulates *brp* expression. (A) Wild-type V. vulnificus cells bearing a *P*_*brpA*_ *lacZ* reporter were grown on LB or marine agar (MA) plates containing X-Gal. (B) Pie charts showing the typical composition of salt in seawater (left) and a breakdown of the concentration (mM) of the major sea salts (right). Data are from the National Oceanographic Data Center (https://www.nodc.noaa.gov/). (C) *P*_*brpA*_ *gfp* expression in V. vulnificus grown in LB or LB supplemented with the major sea salts to concentrations found in seawater. Shown on the right are wild-type V. vulnificus cells bearing a *P*_*brpA*_ *lacZ* reporter growing on LB agar–X-Gal plates containing 50 mM MgCl_2_ (LB^Mg^) or 10 mM CaCl_2_ (LB^Ca^). (D) Fluorescent imaging of V. vulnificus biofilms growing in LB or LB containing 5 or 10 mM CaCl_2_ (LB^Ca-5^ and LB^Ca-10^). An axis indicator is shown in the bottom left corner of each panel. Statistically significant differences among the samples (*P* < 0.001 as determined by one-way ANOVA followed by pairwise comparisons with a Bonferroni adjustment) are indicated by different symbols above each bar.

Our previous investigations indicated that *brpA* expression (monitored using a *P*_*brpA*_ *lacZ* reporter construct) was low in wild-type cells grown in LB. Conversely, the same reporter strain exhibited high-level induction when grown on marine agar (MA) (Fig. 1A), a medium that better reflects the composition of seawater. To identify the component(s) of MA that triggered *brpA* expression, the reporter strain was grown on LB that was systematically supplemented with the major mineral constituents of MA. While the major mono- and divalent cations present in seawater (Fig. 1B) had a negligible effect on *brp* expression when added to LB at typical seawater concentrations, strikingly, the addition of CaCl_2_ to 10 mM strongly induced *P*_*brpA*_ expression (Fig. 1C). These results suggested that calcium specifically regulated *brp* expression. Disruption of either *brpF* or *brpJ* (which encode a glycosyltransferase and a transport protein, respectively) did not affect calcium-induced *brp* expression (see Fig. S1 in the supplemental material), suggesting little or no feedback inhibition of *P*_*brpA*_ expression due to incomplete polysaccharide biosynthesis or failed export of a completed polysaccharide. Notably, while increasing Ca^2+^ concentrations enhanced V. vulnificus biofilm formation (Fig. 1D), it repressed V. cholerae biofilm formation under the same conditions (see Fig. S2 in the supplemental material). These results indicated that environmental calcium can have different effects on biofilm formation by closely related species.

10.1128/mBio.01377-18.1 FIG S1  Calcium-induced *brp* expression is not dependent on Brp production. The indicated wild-type (WT) and *brpF* and *brpJ* mutant strains were grown on LB and LB^Ca^ plates containing X-Gal. Download FIG S1, PDF file, 0.8 MB. Copyright © 2018 Chodur et al. 2018 Chodur et al. This content is distributed under the terms of the Creative Commons Attribution 4.0 International license.

10.1128/mBio.01377-18.2 FIG S2  Inverse effect of calcium on biofilm formation by V. vulnificus and V. cholerae. Wild-type V. vulnificus (Vvu) and V. cholerae (Vch) were grown in LB or LB containing 10 mM CaCl_2_ (LB^Ca^). (Left) Images of the biofilm ring in culture tubes. (Right) Quantification of biofilm formation by crystal violet (CV) staining in 96-well plates. Statistically significant differences among the samples (*P* < 0.001 as determined by one-way ANOVA followed by pairwise comparisons with a Bonferroni adjustment) are indicated by different symbols above each bar. Download FIG S2, PDF file, 0.3 MB. Copyright © 2018 Chodur et al. 2018 Chodur et al. This content is distributed under the terms of the Creative Commons Attribution 4.0 International license.

**FIG 2:**
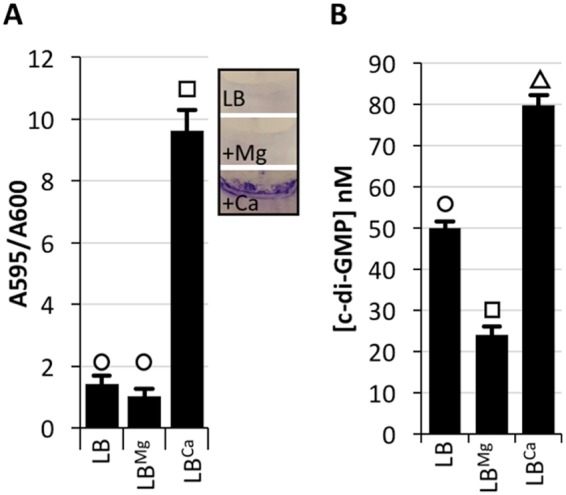
Calcium regulates *brp* expression and biofilm formation by increasing intracellular c-di-GMP levels. (A) V. vulnificus biofilm formation on coverslips during growth in LB or LB supplemented with 50 mM MgCl_2_ (LB^Mg^) or 10 mM CaCl_2_ (LB^Ca^). (B) LC-MS/MS quantification of c-di-GMP from whole-cell extract of V. vulnificus grown in the same medium. Plots show the mean, and error bars represent the standard deviation. Statistically significant differences among the samples (*P* < 0.001 as determined by one-way ANOVA followed by pairwise comparisons with a Bonferroni adjustment) are indicated by different symbols above each bar.

Expression of the *brp* locus is regulated by the intracellular concentration of c-di-GMP (7, 12). To assess if the increase in *brp* expression in response to extracellular calcium correlated with an elevation in intracellular c-di-GMP levels, wild-type cells were grown in LB or LB supplemented with 50 mM MgCl_2_ or 15 mM CaCl_2_ (LB^Mg^ and LB^Ca^, respectively), and biofilm formation and the intracellular c-di-GMP levels were quantified. Biofilm formation was low for cells grown in LB or LB^Mg^ but increased 7-fold in LB^Ca^ (Fig. 2A). The c-di-GMP level in cells that were grown in LB^Mg^ was half that of cells grown in LB (Fig. 2B). Conversely, the c-di-GMP level in cells that were grown in LB^Ca^ was 60% higher than that of cells grown in LB. Collectively, these data suggested that the intracellular c-di-GMP level in V. vulnificus increased in response to environmental calcium, which in turn regulated *brp* expression.

### Cysteine biosynthesis and calcium-induced *brp* expression are genetically linked.

A V. vulnificus transposon mutant library was screened for the loss of *P*_*brpA*_ *lacZ* expression under calcium-inducing conditions to determine how an increase in exogenous calcium concentration resulted in increased *brp* expression (see Table S3 in the supplemental material). Multiple insertions mapped to *brpT*. This was anticipated since *brpT* is absolutely required for *brp* expression (7). Of particular interest was an insertion that mapped to *cysD* (*aot11_12580*), the gene coding for sulfate adenylyltransferase, which participates in cysteine biosynthesis (25). Our previous transcriptome sequencing (RNA-seq) analyses (16) indicated that *cysD* transcript levels increased when intracellular c-di-GMP levels were elevated but failed to do so in a *brpT* mutant. These data suggested that *cysD* expression was positively regulated by c-di-GMP and dependent on BrpT.

**FIG 3:**
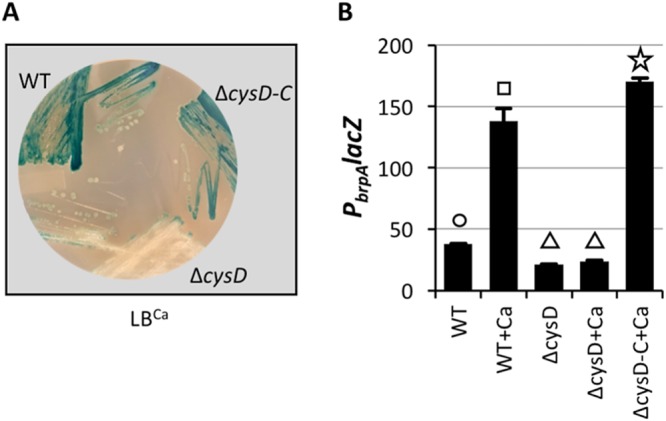
Deletion of *cysD* abrogates calcium-induced *P*_*brpA*_ expression. (A) The wild-type (WT), *cysD* mutant (Δ*cysD*), and complemented strains (Δ*cysD*-*C*) were grown on LB^Ca^ containing X-Gal. (B) The same strains were grown in LB ± 10 mM CaCl_2_ (+Ca) for quantification of *P*_*brpA*_ *lacZ* expression. Statistically significant differences among the samples (*P* < 0.001 as determined by one-way ANOVA followed by pairwise comparisons with a Bonferroni adjustment) are indicated by different symbols above each bar.

A *cysD* null mutant bearing the *P*_*brpA*_ *lacZ* reporter was constructed and grown on LB^Ca^ agar to verify that a functional CysD was indeed required for calcium-induced *brpA* expression. *P*_*brpA*_ expression was elevated in wild-type cells but not in the *cysD* mutant, and this defect could be complemented by expressing an ectopically integrated copy of *cysD* (Fig. 3A). Accordingly, *P*_*brpA*_ *lacZ* expression was low in LB but increased 4-fold in wild-type cells grown in LB^Ca^ (Fig. 3B). In contrast, a mutation in *cysD* prevented calcium induction of *brpA* expression, and this could be complemented by ectopic expression of *cysD*. Thus, a functional CysD was necessary for *P*_*brpA*_ expression in response to calcium.

### The sulfate assimilation metabolic intermediate PAPS mediates calcium-induced *brp* expression.

Disruption of *cysD* is anticipated to render cells auxotrophic for cysteine. However, the Tn libray screen that unearthed *cysD* was conducted on LB agar, which contains up to 0.3 mM cysteine (26). Previous reports of mutant screens on LB agar have resulted in the recovery of cysteine-auxotrophic mutants (27). To determine if the V. vulnificus *cysD* mutant was indeed auxotrophic, the Δ*cysD P*_*brpA*_ *lacZ* reporter strain was grown on minimal medium containing (MM^cys^) or lacking cysteine (MM). The wild-type strain grew well on both types of medium (see Fig. S3 in the supplemental material), while the mutant grew well on MM^cys^ but failed to grow on MM. This confirmed that the *cysD* mutant was indeed auxotrophic for cysteine but that cysteine was not limiting for growth under the conditions used for Tn mutagenesis. Notably, these results suggested that the failure of a *cysD* mutant to induce *brp* expression in response to elevated calcium was unlikely to be rooted in its inability to synthesize cysteine itself. Rather, they implicated an intermediate in cysteine biosynthesis as requisite for calcium-induced *P*_*brpA*_ expression.

10.1128/mBio.01377-18.3 FIG S3  Auxotrophic phenotype of the *cys* mutants. The wild-type (WT) and *cysD*, *cysC*, and *cysH* mutant strains were inoculated onto minimal medium with (MM^cys^) or without (MM) 0.5 mM cysteine. Download FIG S3, PDF file, 0.7 MB. Copyright © 2018 Chodur et al. 2018 Chodur et al. This content is distributed under the terms of the Creative Commons Attribution 4.0 International license.

The *cysDNC* operon participates in assimilatory sulfate activation in bacteria (see Fig. S4 in the supplemental material) (25). Environmental sulfate is transported into the cell, where CysD, together with CysN, then uses ATP and GTP to convert the acquired sulfate to adenosine 5′-phosphosulfate (APS). CysC, an adenylyl-sulfate kinase, uses another molecule of ATP to convert APS to 3′-phosphoadenosine 5′-phosphosulfate (PAPS), better known as activated sulfur. CysH is a sulfotransferase that converts PAPS to sulfite and adenosine 3′,5′-bisphosphate (PAP). Sulfite is converted to sulfide by the action of *cysI* and *cysJ*, which can then participate in sulfurylation reactions or be utilized in cysteine biosynthesis. To identify the sulfate assimilation intermediate regulating *P*_*brpA*_ expression, null mutations that systematically targeted specific steps of *de novo* cysteine biosynthesis were constructed. Deletions of *cysD*, *cysN*, *cysC*, and *cysH* were generated in the *P*_*brpA*_ *lacZ* reporter strain, and the mutants were assayed for growth in medium that included or lacked cysteine. All of the strains grew equally well on MM^cys^, but the mutants failed to grow on MM (Fig. S3), confirming their auxotrophic phenotype.

10.1128/mBio.01377-18.4 FIG S4  Part of the sulfate assimilation pathway leading to cysteine biosynthesis. Environmental sulfate is taken up and converted to adenosine 5′-phosphosulfate (APS) by the action of CysD and CysN. CysC converts APS to 3′-phosphoadenosine 5′-phosphosulfate (PAPS), which is then processed to adenosine 3′,5′-bisphosphate (PAP) and sulfite by CysH. PAP is converted to AMP by CysQ, and sulfite is reduced to sulfide by CysI and CysJ. CysK catalyzes the formation of l-cysteine from sulfide and *O*-acetylserine. Alkane sulfonates that are taken up can directly converted by mono- or dioxygenases to sulfite. Download FIG S4, PDF file, 0.2 MB. Copyright © 2018 Chodur et al. 2018 Chodur et al. This content is distributed under the terms of the Creative Commons Attribution 4.0 International license.

**FIG 4:**
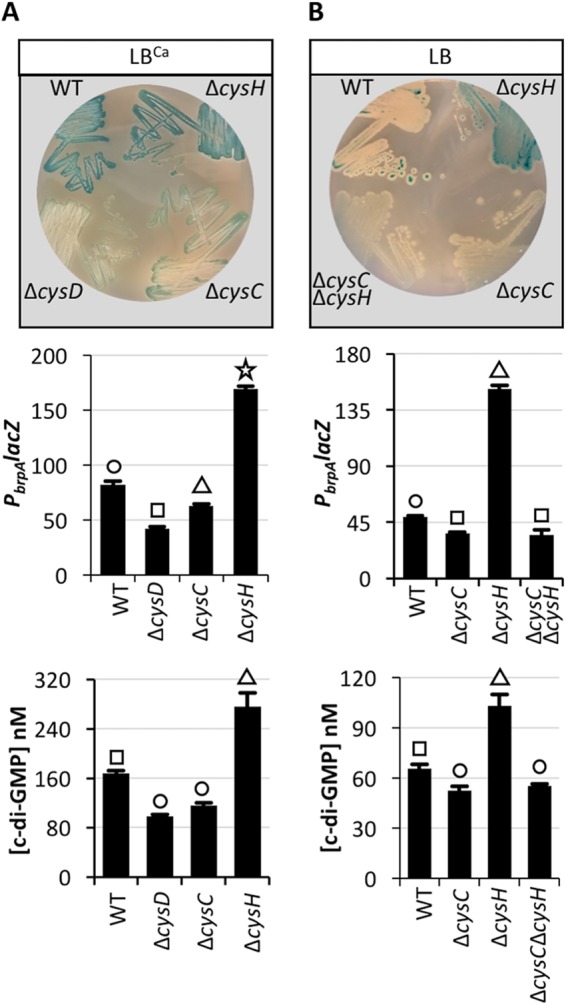
*P*_*brpA*_ expression is reduced in a Δ*cysD* or Δ*cysC* mutant and induced in a *cysH* mutant. The indicated strains were grown on LB^Ca^ (A) or LB (B) agar plates containing X-Gal (top), and the same strains were grown in LB ± 10 mM CaCl_2_ for quantification of *P*_*brpA*_ *lacZ* expression (middle) and the intracellular c-di-GMP concentration (bottom) in the WT and *cys* mutants. Statistically significant differences among the samples (*P* < 0.001 as determined by one-way ANOVA followed by pairwise comparisons with a Bonferroni adjustment) are indicated by different symbols above each bar.

Calcium-induced *P*_*brpA*_ *lacZ* expression was monitored in each mutant background. *P*_*brpA*_ expression in the Δ*cysD*, Δ*cysC*, and Δ*csyH* Δ*cysC* mutants was reduced relative to wild type on LB^Ca^ and LB that lacked calcium (Fig. 4A and B). Conversely, *P*_*brpA*_ expression increased in the Δ*cysH* strain on LB^Ca^ and LB, and cellular c-di-GMP levels of the Δ*cysH* strain were nearly twice that of the wild type and the Δ*cysC*, Δ*cysD*, and Δ*cysC* Δ*cysH* mutants on LB and LB^Ca^. These results suggested that mutations that interfered with the conversion of sulfate to PAPS (Δ*cysD* and Δ*cysC*) inhibited calcium-induced *P*_*brpA*_ expression, while a mutation that favored the accumulation of PAPS by restricting its conversion to sulfite (Δ*cysH*) had increased intracellular c-di-GMP levels and increased *P*_*brpA*_ expression.

### Deletion of *cysD* does not prevent c-di-GMP-mediated *P*_*brpA*_ expression.

**FIG 5:**
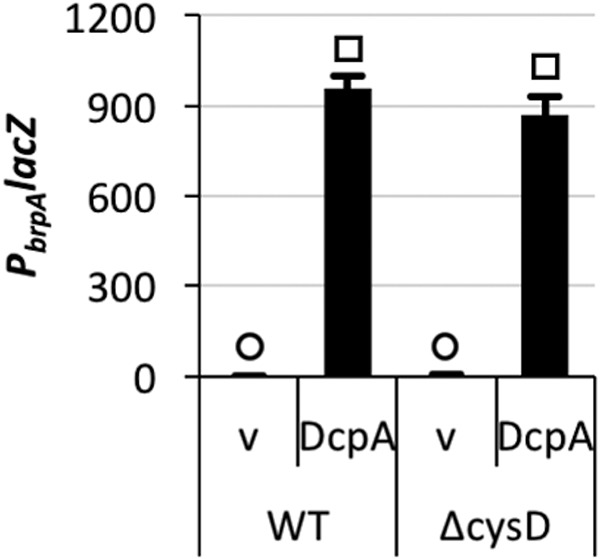
Elevated intracellular c-di-GMP levels alleviate the need for a functional *cysD* for calcium-induced *P*_*brpA*_ expression. *P*_*brpA*_ *lacZ* expression in the wild type and *cysD* mutant under unaltered (v [empty vector]) or elevated (DcpA) intracellular c-di-GMP levels. Statistically significant differences among the samples (*P* < 0.001 as determined by one-way ANOVA followed by pairwise comparisons with a Bonferroni adjustment) are indicated by different symbols above each bar.

To determine if *brp* expression was absolutely dependent on PAPS production, the diguanylate cyclase, DcpA, was expressed in wild-type and Δ*cysD* cells to increase the intracellular c-di-GMP concentration and *P*_*brpA*_ *lacZ* expression was monitored. An equally robust increase in *P*_*brpA*_ expression was observed in both the wild-type and Δ*cysD* strains (Fig. 5). This suggested that elevated intracellular c-di-GMP levels could bypass the input of calcium, *cysD*, and PAPS in regulating *brp* expression.

### PAPS production is required for rugosity.

**FIG 6:**
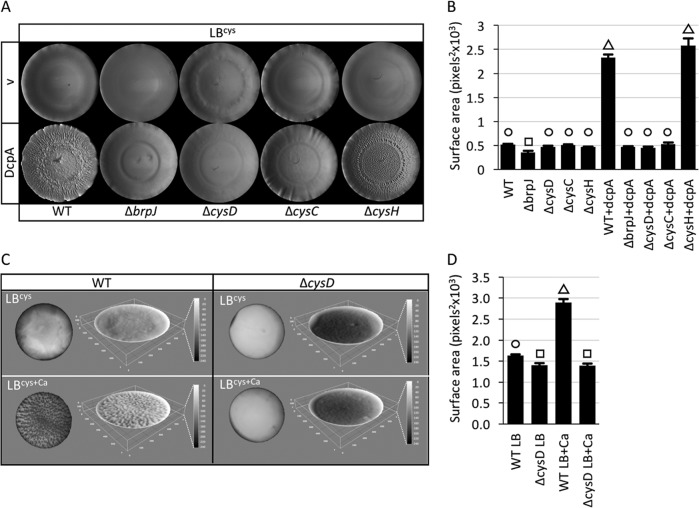
Mutations in *cysC* and *cysD* inhibit rugose colony formation in response to elevated intracellular c-di-GMP levels and calcium-induced pellicle formation. (A) Rugose colony development by the indicated strains under unaltered (v [empty vector]) or elevated (DcpA) intracellular c-di-GMP levels on LB agar plates supplemented with 0.5 mM cysteine (LB^cys^). (B) Quantification of the surface area of the rugose colonies in panel A. (C) The wild-type (WT) and *cysD* mutant strains were grown in LB^cys^ or LB^cys+Ca^, and pellicle formation at the air-liquid interface was imaged. The left image in each panel is the top-down view of the pellicle, and to the right is the 3D rendering, where the dark-to-light transition indicates low-lying to elevated surface area regions. The scale is to the right in each panel. (D) Quantification of the pellicle surface area in panel C.

The extent of rugose colony development in response to increased intracellular c-di-GMP concentrations in the wild-type and Δ*cysD* strains did not correlate with their comparably equal levels of *P*_*brpA*_ expression. Rugose colony formation and an associated increase in surface area were evident in wild-type cells expressing DcpA (Fig. 6A and B). A lesion in *brpJ* abrogated this phenotype, indicating that rugose colony development was dependent on the production of the *brp* polysaccharide. While there was little effect on rugose colony development when *cysH* was deleted, the Δ*cysD* and Δ*cysC* strains failed to become rugose when intracellular c-di-GMP levels were increased. Moreover, *P*_*brpA*_ expression increased in a Δ*cysD* background when intracellular c-di-GMP levels were elevated (Fig. 5), yet colonies failed to become rugose, and deletion of *cysD* abrogated pellicle formation at the air-liquid interface relative to wild-type cells (Fig. 6C and D). These results suggested that the ability to produce PAPS (which is reduced in Δ*cysD* and Δ*cysC* relative to wild-type and Δ*cysH* cells) was required for rugose colony development and likely pellicle formation in a manner that was distinct from its role in regulating *P*_*brpA*_ expression.

### c-di-GMP and BrpT regulate the expression of genes in the sulfate assimilation pathway.

**FIG 7:**
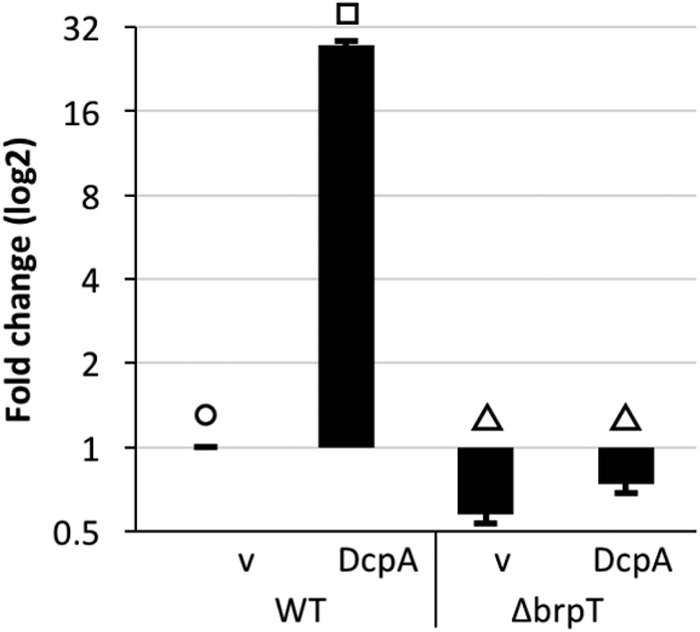
The expression of *cysD* is c-di-GMP and BrpT dependent. qRT-PCR was used to determine *cysD* transcript levels in wild-type (WT) and Δ*brpT* cells under unaltered (v [empty vector]) and elevated (DcpA) intracellular c-di-GMP levels. Expression values are relative to those of WT under unaltered conditions. Statistically significant differences among the samples (*P* < 0.001 as determined by one-way ANOVA followed by pairwise comparisons with a Bonferroni adjustment) are indicated by different symbols above each bar.

Our previous comparative transcriptomic analyses under unaltered and elevated cellular c-di-GMP conditions (12) suggested two groupings for the genes of sulfate assimilation: the expression of those involved in the conversion of sulfate to PAPS (*cysDNC*) increased when intracellular c-di-GMP concentrations were elevated, while those required for the production of cysteine from PAPS (*cysHIJ*) were downregulated (see Table S4 in the supplemental material). Moreover, the expression of *cysDNC* appeared to be positively regulated by BrpT. To confirm this, quantitative reverse transcription-PCR (qRT-PCR) was performed on RNA isolated from wild-type and Δ*brpT* cells expressing DcpA or carrying the empty vector. In agreement with our RNA-seq data, the expression of *cysD* was strongly induced in wild-type cells under elevated intracellular c-di-GMP conditions (Fig. 7), while the expression of *cysJ* decreased 2-fold (see Fig. S5 in the supplemental material). Moreover, the c-di-GMP-mediated increase in *cysD* expression was negated in a *brpT* mutant, indicating that *cysD* is positively regulated by c-di-GMP and BrpT. Thus, not only does the production of PAPS trigger *brp* expression, but the activity of BrpT, the transcriptional activator of *brp* expression, biases the sulfur assimilation pathway to favor the accumulation of PAPS (i.e., the expression of genes directing PAPS production is increased while the expression of genes that would deplete the PAPS pool is repressed).

10.1128/mBio.01377-18.5 FIG S5  The expression of *cysJ* is c-di-GMP-dependent. qRT-PCR was used to confirm that *cysJ* transcript levels decreased in wild-type cells when intracellular c-di-GMP levels were elevated (DcpA) relative to unaltered (v [empty vector]) conditions. Expression values are relative to those under unaltered conditions. Statistical significance was determined by the Student *t* test (two-tailed distribution with two-sample, equal variance). Download FIG S5, PDF file, 0.1 MB. Copyright © 2018 Chodur et al. 2018 Chodur et al. This content is distributed under the terms of the Creative Commons Attribution 4.0 International license.

## DISCUSSION

c-di-GMP regulates the expression of the *brp*-encoded extracellular polysaccharide that promotes V. vulnificus biofilm formation and rugose colony development (7). Temperature is one of the few primary environmental signals known to affect *brp* expression in V. vulnificus (12). Here, we identify calcium as a primary signal regulating biofilm formation in V. vulnificus. Calcium, second to magnesium in divalent abundance in seawater (28), specifically triggered *brp* expression. Genetic screening revealed that an intermediate of the sulfate assimilation pathway (PAPS) was part of the regulatory circuitry altering *brp* expression in response to changes in environmental calcium concentration. Our genetic data suggested that genes required for the production of PAPS in response to extracellular calcium affected *brp* expression in V. vulnificus. Mutation of *cysD*, *cysC*, or *cysH* is known to alter the intracellular level of the PAPS intermediate in other bacteria (29–33). We showed that *cysD* and *cysC* null mutants, which cannot produce PAPS, could not induce *P*_*brpA*_ in response to increased environmental calcium, whereas a Δ*cysH* mutant, which accumulates PAPS, exhibited increased *P*_*brpA*_ expression even in the absence of added calcium. Although we did not directly quantify intracellular PAPS levels (the modified nucleotide is highly unstable) (34), intracellular c-di-GMP (and presumably PAPS) levels are elevated in a V. vulnificus *cysH* mutant relative to the wild type. Thus, mutations in genes that decreased (*cysD* and *cysC*) or increased (*cysH*) PAPS production influenced calcium-induced *brp* expression. Moreover, elevation of intracellular c-di-GMP levels increased the expression of genes required for PAPS production (*cysDNC*), and this increase was BrpT dependent. c-di-GMP also repressed the expression of *cysHIJ*, the products of which drain the PAPS pool. Thus, this feed-forward signaling network biased the sulfur metabolism pathway toward PAPS accumulation, which in turn increased cellular c-di-GMP levels, which increased *brpT* expression, which ultimately increased production of both Brp polysaccharide and PAPS. Disruption of *brpF* or *brpJ* (which encode a glycosyltransferase and a transport protein, respectively) did not affect calcium-induced *brp* expression, suggesting little or no feedback inhibition of *P*_*brpA*_ expression due to incomplete polysaccharide biosynthesis or failed export of a completed polysaccharide. It was recently reported that lesions in *cysH*, *cysJ*, *cysK*, and *cysN* negatively impacted rugose colony development in Vibrio fischeri (35). However, the defect was most severe in the *cysK* mutant, while disruption of *cysN* had little impact, suggesting that the mechanism must differ from that in V. vulnificus.

A second messenger is an intracellular signaling molecule whose concentration increases or decreases in response to the binding of an extracellular ligand to a cell surface receptor. Thus, PAPS behaves as a second messenger in V. vulnificus, akin to c-di-GMP. We propose that elevated environmental calcium increases PAPS production, which ultimately mediates *brp* expression by altering the cellular c-di-GMP pool. PAPS production may regulate *eps* production in Vibrio parahaemolyticus as well; lesions in *cysD* and *cysC* prevented pellicle formation at the air-liquid interface, a phenotype that is dependent on expression of the *cps* polysaccharide locus (36). A V. vulnificus *cysD* mutant exhibited the same defect. This is noteworthy since V. vulnificus and V. parahaemolyticus occupy similar environmental niches (37). Our data suggested that PAPS levels also affected the production of a biofilm matrix component, in an addition to the Brp polysaccharide, that is essential for rugosity. A candidate is the calcium-binding matrix protein CabA, which is required for biofilm and rugose colony formation (17). It was recently demonstrated that *cabABC* expression in V. vulnificus is c-di-GMP and BrpT dependent (16). Although elevation of the cellular c-di-GMP level (conditions under which CabA should be produced) overcame the calcium-induced *P*_*brpA*_ expression defect of the V. vulnificus *cysD* and *cysC* mutants, it was not sufficient to support rugose colony development. PAPS may influence CabA activity, or PAPS may regulate the production of another unidentified matrix component.

APS and PAPS are modified nucleotide intermediates of sulfate assimilation that have both been shown to have regulatory effects on gene expression. In Escherichia coli, APS acts as a signaling molecule that is directly bound by the transcriptional regulator Cbl. This results in the inhibition of genes required for organosulfonate utilization and thus helps to establish the hierarchy of preferred sources of sulfur (38). Expression of the *csgBAC* locus that governs curli production in E. coli is controlled by the regulator CsgD (39), and *csgD* expression is regulated by the PDE YciR (40). Hence, *csgBAC* expression is c-di-GMP dependent. It was recently demonstrated that *csgBAC* expression increased in a Δ*cysH* strain, while a Δ*cysD* Δ*cysH* double mutant or overexpression of *cysH* decreased *csgBAC* transcription (33). Since, *csgBAC* expression is affected by the intracellular concentration of both c-di-GMP and PAPS, and *csgBAC* expression increased on a Δ*cysH* strain, this suggests that PAPS accumulation must lead to altered cellular c-di-GMP concentrations and suggests that the signaling link we identified between the two second messengers in V. vulnificus may be evolutionarily conserved in E. coli and potentially other Gram-negative bacteria. Ultimately, PAPS appears to mediate the expression of genes for cell surface modification in response to environmental stimuli (curli in E. coli and EPS in V. vulnificus and V. parahaemolyticus). Our study clearly demonstrates interplay between PAPS and c-di-GMP signaling in V. vulnificus and PAPS was recently shown to impact cAMP receptor protein (CRP)-cAMP regulatory networks in E. coli (41), suggesting overlap in the pathways regulated by these three modified nucleotides (cAMP, c-di-GMP, and PAPS). Collectively, these studies highlight the widening regulatory influence of PAPS on bacterial physiology and its intersection with other modified nucleotide-regulated pathways positions PAPS as a potential global signaling molecule.

**FIG 8:**
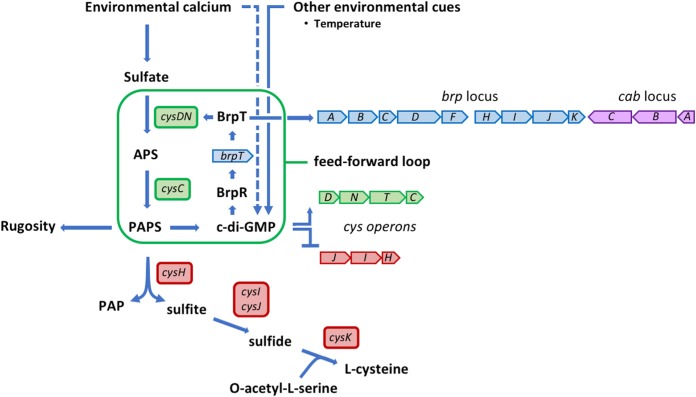
Model for the integration of environmental calcium signaling and PAPS production in the regulation of *brp* expression. BrpT and c-di-GMP bias the sulfate assimilation pathway toward the accumulation of PAPS in a feed-forward regulatory network (framed in green). Points of activation and repression are indicated by solid blue arrowheads and flat lines, respectively. Environmental calcium stimulates sulfate uptake. The sulfate is converted to adenosine 5′-phosphosulfate (APS) by the action of CysD and CysN and converted to 3′-phosphoadenosine 5′-phosphosulfate (PAPS) by CysC. The accumulation of PAPS triggers an increase in the intracellular c-di-GMP level, which increases *brpT* expression via the regulator BrpR and also represses expression of the *cysJIH* operon that drains the PAPS pool by processing it to PAP and sulfite and, eventually, to cysteine. BrpT activates expression of the *brp* exopolysaccharide, *cab* matrix genes, and *cysDNTC* operons (blue, purple, and green block arrows, respectively), which generates more PAPS, c-di-GMP, and BrpT, thus establishing a feed-forward signaling network that biases the sulfur metabolism pathway toward accumulating PAPS to increase *brp* expression and biofilm formation. PAPS also has a separate and distinct impact on rugose colony development. The operon structure of *cysDNTC* and *cysJIH* (red block arrow) allows for their simultaneous regulation by c-di-GMP and, for the regulation of *cysDNTC*, by BrpT. Environmental calcium may also influence cellular c-di-GMP levels independent of sulfate assimilation (dashed blue line). Other environmental cues (e.g., temperature) can directly affect intracellular c-di-GMP concentrations that may then feed into the PAPS–c-di-GMP loop.

We propose the following model for the integration of environmental calcium signaling and PAPS production in the regulation of *brp* expression (Fig. 8). Environmental calcium upregulates sulfate assimilation. Sulfate that is taken up is converted to APS by the action of CysD and CysN and converted to PAPS by CysC. The accumulation of PAPS triggers an increase in the intracellular c-di-GMP level, which in turn increases *brpT* expression via the regulator BrpR, while simultaneously repressing expression of the *cysJIH* operon that normally drains the PAPS pool by processing it into sulfide and eventually cysteine. BrpT activates expression of the *brp* exopolysaccharide and *cysDNTC* operons, which generates more PAPS, c-di-GMP, and BrpT. Thus, a feed-forward regulatory network is established in which calcium, c-di-GMP, and BrpT bias the sulfate assimilation pathway toward the accumulation of PAPS and increased *brp* expression.

Oysters and other bivalves have the capacity to locally concentrate calcium for shell growth and repair (42). Conceptually, the ability of V. vulnificus to sense a change in environmental calcium concentration and initiate biofilm formation at the transcriptional level makes it a perfect cue to promote niche (oyster) colonization by this pathogen. Calcium can repress *Vibrio* polysaccharide (VPS)-dependent biofilm formation by V. cholerae (43), although V. cholerae can also form distinct Ca^2+^-dependent/VPS-independent biofilms in which calcium is proposed to interact with negatively charged groups in the O antigen or capsule and physically mediate surface adhesion (44, 45). The different physiological biofilm responses of V. vulnificus and V. cholerae to calcium under certain conditions may help explain how these closely related species use similar biofilm networks yet evolved to occupy distinct niches in a shared environment. The adoption of calcium as a primary signaling molecule for V. vulnificus biofilm formation is ideal, and it is intriguing to think that V. vulnificus may have in fact evolved to exploit calcium as a molecular beacon to indicate preferred environments for colonization.

## MATERIALS AND METHODS

### Media and strains.

LB, LB agar, Bacto agar, and 2216 marine broth were purchased from BD Difco. Marine agar plates contained 1.5% Bacto agar. Antibiotics and additives were purchased from Sigma and used at the following concentrations: ampicillin, 100 µg/ml; chloramphenicol and gentamicin, 35 µg/ml; isopropyl-β-d-thiogalactopyranoside (IPTG), 100 µM; X-Gal (5-bromo-4-chloro-3-indolyl-β-d-galactopyranoside), 100 µg/ml; and l-arabinose, 0.05%. Minimal medium plates contained 40 mM HEPES (pH 7.2), 0.4% galactose, 1× M9 salts, 0.02% Difco yeast extract, and 1.5% Bacto agar. Strains are listed in Table S1 in the supplemental material.

10.1128/mBio.01377-18.6 TABLE S1  Strains and plasmids used in this study. Download TABLE S1, DOCX file, 0.1 MB. Copyright © 2018 Chodur et al. 2018 Chodur et al. This content is distributed under the terms of the Creative Commons Attribution 4.0 International license.

### Fluorescent reporter fusion analysis.

The wild-type strain bearing the *P*_*brpA*_ *gfp* reporter was grown overnight, diluted 100-fold in fresh medium containing the indicated salts, and inoculated in triplicate into a 96-well plate. After 16 h at 30°C, the optical density at 600 nm (OD_600_) and florescence measurements were read on a BioTek Synergy H1 microplate reader (excitation, 485 nm; emission, 528 nm). Data of three technical replicates from three biological replicates were collected for all experiments. Plots show the mean, and error bars represent the standard deviation.

### β-Galactosidase assays.

Quantification of β-galactosidase activity was carried out following the original Miller protocol (46) with the following amendments. Briefly, 1 OD_600_ unit of cells was pelleted by centrifugation for 10 min at 12,000 × *g*. Cell pellets were resuspended in 1 ml Z buffer (0.06 M Na_2_HPO_4_, 0.04 M NaH_2_PO_4_, 0.01 M KCl, 0.001 M MgSO_4_, 0.05 M β-mercaptoethanol, pH 7.0). Next, 50 µl on 1% SDS was added and the mixture was vortexed. Chloroform (100 µl) was added, and the sample was vortexed for 10 s and then centrifuged for 2 min at 12,000 × *g*. A 150-µl aliquot of each sample was transferred to the well of a 96-well plate containing 50 µl (10 mg/ml) of *o*-nitrophenyl-β-d-galactopyranoside (ONPG) and placed in a BioTek Synergy HT microplate reader, where *A*_420_ and *A*_595_ measurements were taken in 5-min intervals. Modified Miller units were calculated using the formula described in the original assay. All assays were performed in triplicate.

### Tn mutagenesis.

The mini-Tn*10* delivery vector pNKTXI-SceI (47) was used for transposon mutagenesis. Cultures of V. vulnificus bearing a *P*_*brpA*_ *lacZ* reporter and E. coli bearing our Tn on a replicating plasmid were grown at 30 and 37°C, respectively. After overnight growth, cultures were diluted by 1:100 and grown to an OD_600_ of 0.5. One milliliter of each culture was washed of antibiotics by pelleting with centrifugation and resuspended in 100 µl of LB. The E. coli-V. vulnificus mixture was then placed on a nitrocellulose filter disc on an LB plate and placed at 37°C for conjugation. After overnight growth, cells from the filter disc were resuspended in 5 ml LB and plated onto LB with rifampin, 120 µg/ml X-Gal, 2 µg/ml chloramphenicol, 160 µg/ml kanamycin, and 15 mM CaCl_2_. After 48 h of growth, white colonies (representing loss of *brp* expression in the presence of calcium) were rescreened on plates containing a 10 mM mixture of rifampin, X-Gal, chloramphenicol, kanamycin, and CaCl_2_. A total of 30,000 mutants were screened on LB^Ca^ plates containing X-Gal for isolates exhibiting decreased *P*_*brpA*_ *lacZ* expression. Identified mutants were pooled, and collective genomic DNA was prepared and subjected to whole-genome sequencing at the Indiana University—Bloomington Center for Genomics and Bioinformatics (IUB-CGB). Geneious (Biomatters) was used to map the reads to the mini-Tn*10* reference sequence and recover flanking V. vulnificus genomic sequences that were mapped back against the whole genome of strain ATCC 27562 (19). In-frame markerless deletions of target genes were generated by splicing by overhang extension (SOE) PCR (see below).

### Mutant construction.

Allelic replacement of the target gene was achieved by SOE PCR as follows. PCR fragments corresponding to 3 kb upstream and downstream of the target gene (for primers, see Table S2 in the supplemental material) were amplified and stitched to a central trimethoprim or kanamycin antibiotic resistance cassette. Recipient strains carrying the pMMB-TfoX expression plasmid (48) were induced overnight in LB containing ampicillin and IPTG at 30°C. A 10-µl aliquot of cells was added to 500 µl of Instant Ocean at 20 ppt (IO-20) containing IPTG and 25 µl of the SOE PCR product. The transformation mixture was incubated statically overnight at 30°C. The next day, 1 ml of LB was added to the tube, and the cells were allowed to outgrow for 6 h before being plated on LB with selective antibiotics. Allelic replacement of the target gene was confirmed by PCR.

10.1128/mBio.01377-18.7 TABLE S2  Primers used in this study. Download TABLE S2, DOCX file, 0.2 MB. Copyright © 2018 Chodur et al. 2018 Chodur et al. This content is distributed under the terms of the Creative Commons Attribution 4.0 International license.

### Rugose colony morphology assays and crystal violet staining of biofilms.

For visualization of *P*_*brpA*_ *lacZ* expression, strains were grown overnight on LB plates that included the appropriate antibiotics, supplements, and X-Gal at 30°C. Images were captured after 48 h using a Leica MS5 dissecting scope equipped with a Leica DC300F camera. To assess rugose colony morphology, 2-µl aliquots of overnight cultures were spotted onto the indicated LB plates and incubated at 30°C for overnight, followed by an additional day at room temperature. Colony morphology images were captured as described above. A fixed radius was used to determine the surface area for colonies with the SP package (49) in R (50). Corresponding 3D elevation maps were created with ImageJ (51). A representative image of samples done in triplicate is shown.

For crystal violet staining, plastic coverslips were placed into 12-well tissue culture plates containing 3 ml of LB medium plus amendments and inoculated with an exponentially growing culture of the indicated strain to an OD_600_ of 0.025. After 16 h of static growth at 30°C, coverslips were removed and placed into 4 ml of 0.1% crystal violet for 10 min to stain. The coverslips were rinsed 3 times in 4 ml VPBS (130 mM NaCl, 5 mM Na_2_HPO_4_, 1.5 mM KH_2_PO_4_, pH 7.4 [52]), and the violet stain was solubilized in 4 ml of 4:1 isopropanol-acetone. The *A*_595_ reading was normalized to the OD_600_ for each sample. All assays were done in triplicate.

### qRT-PCR.

The primers used to monitor expression of the indicated genes are listed in Table S2. Strains carrying an arabinose-inducible plasmid expressing the DGC *dcpA* (7) were grown with antibiotics overnight at 30°C. The next morning, an aliquot was diluted 1:100 into fresh medium and grown to an OD_600_ of 0.5. The culture was then diluted into 5 ml of LB containing 0.1% arabinose (to induce DcpA expression) to an OD_600_ of 0.025 and grown for 5 h at 30°C. RNA was extracted from 1 OD unit of pelleted cells using the Bio Basic Total RNA miniprep kit. qRT-PCR was carried out using the SensiFAST SYBR Hi-ROX one-step kit. Reactions were performed on an Applied Biosystems StepOnePlus real-time PCR system. Data were analyzed using double threshold cycle (Δ*C*_*T*_) analysis. All assays were done in triplicate.

### Monitoring biofilm development in microfluidic chambers.

Polydimethylsiloxane (PDMS)-glass flow cell devices containing eight 40- by 5- by 1-mm chambers were fabricated and sterilized (53). Mid-log-phase wild-type cells (OD_600_ of 0.1) were seeded into separate flow cell chambers. Control chambers for background fluorescence contained medium only. Initial attachment (no flow) proceeded for 20 min, followed by a flow rate of 0.25 ml min^−1^ in LB or LB supplemented with 5 or 10 mM CaCl_2_ for 16 h. The chambers were then flooded with the same medium containing 2.5 µM Syto21 (Molecular Probes) for 10 min. Biofilm images and z-stacks (20 × 1-µm slices) were captured with an Olympus IX83 microscope using a UPLSAPO 40× silicon oil immersion objective (NA, 1.25; WD, 0.3 mm). Background fluorescence was subtracted from each sample, and quantitative analysis to determine biomass was performed using cellSense (Olympus) and Comstat (54). Data from three biological replicates were analyzed for each strain. The images presented are from a single representative experiment.

### Quantification of the intracellular c-di-GMP concentration.

c-di-GMP was extracted from whole cells as previously described (12). Briefly, the indicated strains were grown overnight in LB or LB^Ca^ with antibiotics when appropriate. The next day, cells were diluted 1:100 in fresh medium and grown to an OD_600_ of 0.5. Cells were again diluted to an OD_600_ of 0.025 into fresh LB supplemented with the indicated cations and l-Ara where indicated. Cells were grown for 12 h, when peak *brp* expression was observed in the presence of CaCl_2_. Six OD units of each sample was centrifuged at 4°C for 10 min at 12,000 × *g*. The pellet was resuspended in 200 µl of 40% (vol/vol) acetonitrile–40% (vol/vol) methanol–20% 0.1 N formic acid. This suspension was incubated at −20°C for 30 min and then centrifuged at 4°C for 5 min. The supernatant was recovered, and 10 µl of each sample was analyzed by liquid chromatography-tandem mass spectrometry (LC-MS/MS) on a Quattro Premier XE mass spectrometer (Waters) coupled with an Acquity Ultra Performance LC system (Waters). c-di-GMP was detected by electrospray ionization using multiple reaction monitoring in negative-ion mode at *m*/*z* 689.16→344.31. The MS parameters were as follows: capillary voltage, 3.5 kV; cone voltage, 50 V; collision energy, 34 V; source temperature, 110°C; desolvation temperature, 350°C; cone gas flow (nitrogen), 50 liters/h; desolvation gas flow (nitrogen), 800 liters/h; collision gas flow (nitrogen), 0.15 ml/min; and multiplier voltage, 650 V. Chromatography separation was reverse phase using a Waters BEH C_18_ 2.1- by 50-mm column at a flow rate of 0.3 ml/min with a gradient of 10 mM tributylamine plus 15 mM acetic acid in 97:3 water-methanol (solution A) to methanol (solution B) with the following parameters: *t* = 0 min; 99% A:1% B, *t* = 2.5 min; 80% A:20% B, *t* = 7.0 min; 35% A:65% B, *t* = 7.5 min; 5% A:95% B, *t* = 9.01 min; and 99% A:1% B, *t* = 10 min. A c-di-GMP standard curve for calculating the c-di-GMP concentration in each extract was generated by dissolving synthesized c-di-GMP (Axxora) in extraction buffer to 250 nM, followed by 2-fold serial dilutions to 0.975 nM. Data from three biological replicates were analyzed for each strain.

### Statistical analyses.

Statistical significance was determined by the Student *t* test (two-tailed distribution with two-sample, equal variance) when directly comparing two conditions or a one-way analysis of variance (ANOVA) followed by pairwise comparisons with a Bonferroni adjustment when comparing data with multiple samples.

10.1128/mBio.01377-18.8 TABLE S3  Tn insertions that disrupt calcium-induced *P_brpA_* expression. Download TABLE S3, DOCX file, 0.1 MB. Copyright © 2018 Chodur et al. 2018 Chodur et al. This content is distributed under the terms of the Creative Commons Attribution 4.0 International license.

10.1128/mBio.01377-18.9 TABLE S4  Sulfate assimilation pathway genes regulated by BrpT and c-di-GMP. Download TABLE S4, DOCX file, 0.1 MB. Copyright © 2018 Chodur et al. 2018 Chodur et al. This content is distributed under the terms of the Creative Commons Attribution 4.0 International license.
