# Supplementary material for: Environmental Calcium Initiates a Feed-Forward Signaling Circuit That Regulates Biofilm Formation and Rugosity in Vibrio vulnificus
Source: mBio. 2018 Aug 28;9(4):e01377-18. doi: 10.1128/mBio.01377-18 (PMC6113621; doi:10.1128/mBio.01377-18)
Supplement: FIG S1 [file mbo004184044sf1.pdf]

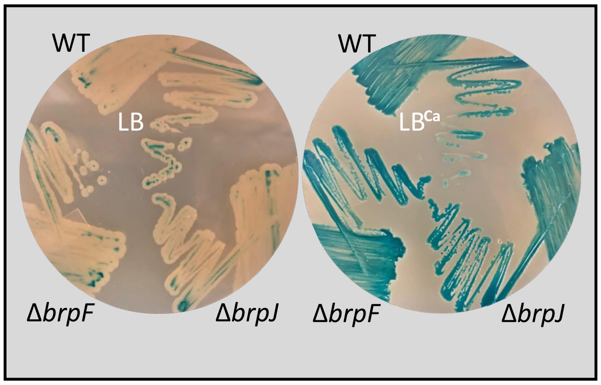

Figure S1. Calcium-induced *brp* expression is not dependent on BRP production. The indicated wildtype (WT), *brpF* and *brpJ* mutant strains were were grown on LB and LB^{Ca} plates containing X-gal.
