# Supplementary material for: Environmental Calcium Initiates a Feed-Forward Signaling Circuit That Regulates Biofilm Formation and Rugosity in Vibrio vulnificus
Source: mBio. 2018 Aug 28;9(4):e01377-18. doi: 10.1128/mBio.01377-18 (PMC6113621; doi:10.1128/mBio.01377-18)
Supplement: FIG S2 [file mbo004184044sf2.pdf]

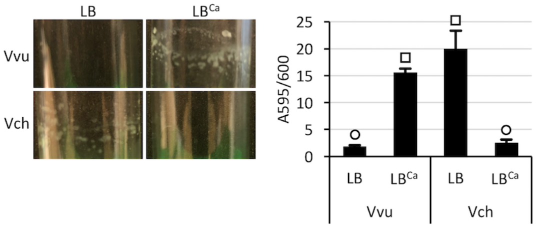

Figure S2. Inverse effect of calcium on biofilm formation by *V. vulnificus* and *V. cholerae*. Wildtype *V. vulnificus* (Vvu) and *V. cholerae* (Vch) were were grown in LB or LB containing 10 mM $CaCl_2$ (LB^{Ca}). Left, images of the biofilm ring in culture tubes. Right, quantification of biofilm formation by CV staining in 96-well plates. Statistically significant differences among the samples ($p < 0.001$ as determined by one-way ANOVA followed by pairwise comparisons with a Bonferroni adjustment) are indicated by different symbols above each bar.
