# Supplementary material for: Environmental Calcium Initiates a Feed-Forward Signaling Circuit That Regulates Biofilm Formation and Rugosity in Vibrio vulnificus
Source: mBio. 2018 Aug 28;9(4):e01377-18. doi: 10.1128/mBio.01377-18 (PMC6113621; doi:10.1128/mBio.01377-18)
Supplement: FIG S3 [file mbo004184044sf3.pdf]

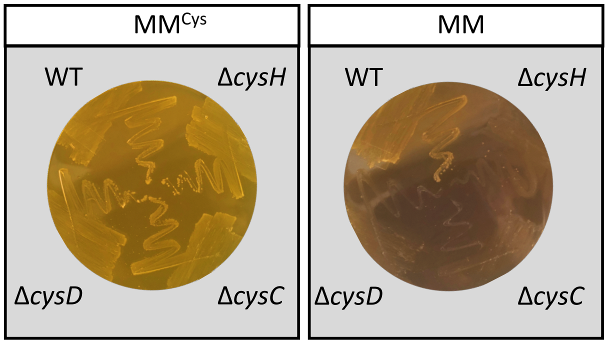

Figure S3. Auxotrophic phenotype of the *cys* mutants. The wildtype (WT), *cysD*, *cysC* and *cysH* mutants were inoculated onto minimal media (MM) with or without 0.5 mM cysteine (MM^{cys}).
