# Supplementary material for: Environmental Calcium Initiates a Feed-Forward Signaling Circuit That Regulates Biofilm Formation and Rugosity in Vibrio vulnificus
Source: mBio. 2018 Aug 28;9(4):e01377-18. doi: 10.1128/mBio.01377-18 (PMC6113621; doi:10.1128/mBio.01377-18)
Supplement: FIG S4 [file mbo004184044sf4.pdf]

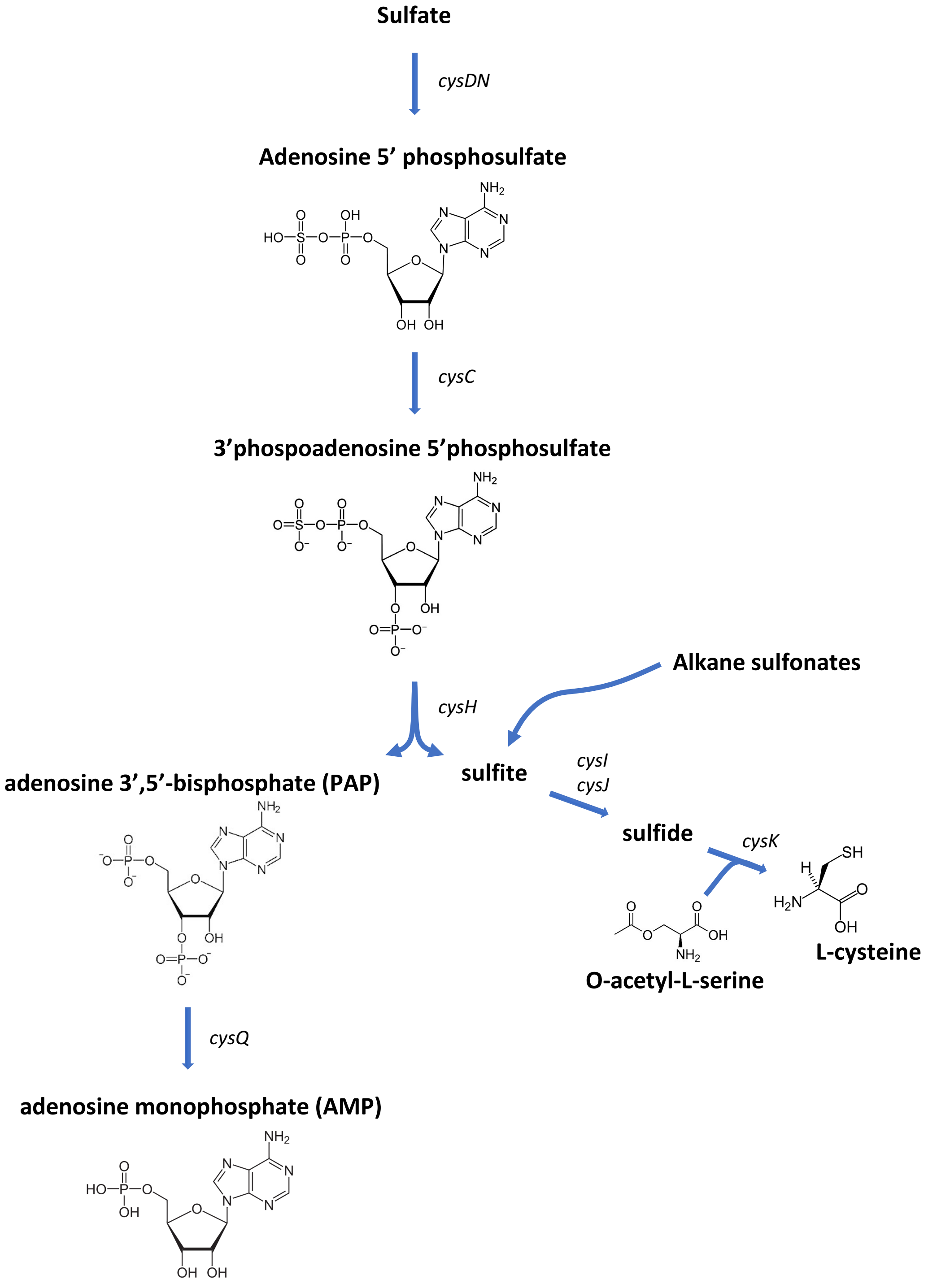

Figure S4. Part of the sulfate assimilation pathway leading to cysteine biosynthesis. Environmental sulfate is taken up and converted to adenosine 5' phosphosulfate (APS) by the action of CysD and CysN. CysC converts APS to 3'phospoadenosine 5'phosphosulfate (PAPS) that is then processed to adenosine 3',5'-bisphosphate (PAP) and sulfite by CysH. PAP is converted to adenosine monophosphate (AMP) by CysQ, and sulfite is reduced to sulfide by CysI and CysJ. CysK catalyzes the formation of L-cysteine from sulfide and O-acetylserine. Alkane sulfonates that are taken up can directly converted by mono or dioxygenases to sulfite.
