# Supplementary material for: Environmental Calcium Initiates a Feed-Forward Signaling Circuit That Regulates Biofilm Formation and Rugosity in Vibrio vulnificus
Source: mBio. 2018 Aug 28;9(4):e01377-18. doi: 10.1128/mBio.01377-18 (PMC6113621; doi:10.1128/mBio.01377-18)
Supplement: FIG S5 [file mbo004184044sf5.pdf]

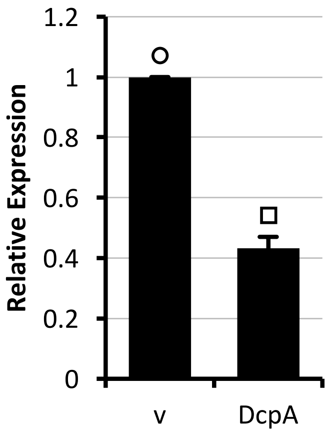

Figure S5. The expression of *cysJ* is c-di-GMP dependent. qRT-PCR was used to confirm that *cysJ* transcript levels decreased in wildtype cells when intracellular c-di-GMP levels were elevated (DcpA) relative to unaltered (v) conditions. Expression values are relative to those under unaltered conditions. Statistical significance was determined by the Student t-test (two-tailed distribution with two-sample, equal variance).
