# Supplementary material for: Environmental Calcium Initiates a Feed-Forward Signaling Circuit That Regulates Biofilm Formation and Rugosity in Vibrio vulnificus
Source: mBio. 2018 Aug 28;9(4):e01377-18. doi: 10.1128/mBio.01377-18 (PMC6113621; doi:10.1128/mBio.01377-18)
Supplement: TABLE S1 [file mbo004184044st1.docx]

**Table S1. Strains and plasmids used in this study.**

| **Strain** | **Genotype/Purpose** | **Ref** |
| --- | --- | --- |
| ***Vibrio vulnificus*** | ATCC 27562 | ATCC |
|  | *ΔbrpT*; marker-less deletion | (1) |
|  | *ebgA::P_brpA_lacZEc* | This study |
|  | *ebgA::P_brpA_lacZEcΔcysD::TmR* | This study |
|  | *ebgA::P_brpA_lacZEcΔcysC::TmR* | This study |
|  | *ebgA::P_brpA_lacZEcΔcysH::TmR* | This study |
|  | *ebgA::P_brpA_lacZEcΔcysN::TmR* | This study |
|  | *ebgA::P_brpA_lacZEcΔcysC::kanΔcysH::TmR* | This study |
|  | *ebgA::P_brpA_lacZEcΔbrpJ* | This study |
|  | *ebgA::P_brpA_lacZEcΔbrpF* | This study |
| ***Vibrio cholerae*** | O1 biovar El Tor N16961 | ATCC |
|  |  |  |
| ***E. coli*** | S17.1λπ ; RP4 conjugation machinery, *pir* | (2) |
|  | BL21(DE3); IPTG-inducible T7 expression | NEB |
| **Plasmids** |  |  |
| pC2X6HIST | Expression vector (1) | (1) |
| pC2X6HIST::cysD | Expression of CysD | This study |
| pSU38GTAraC | Expression vector (1) | (1) |
| pSU38GTaraC::*dcpA* | Expression of DcpA | (1) |
| pTX1K*lacZA^Vv^* | Integration into the chromosomal *lacZ* of *V. vulnificus* | This study |
| pTX1K*lacZ^Vv^::P_brpA_lacZ^Ec^* | Integration of *P_brpA_lacZ^Ec^* into the chromosomal *lacZ* | This study |
| pSW23T*ebgA* | Integration into chromosomal *ebgA* (cryptic *lacZ*) | This study |
| pSW23T*ebgA*::*P_brpA_gfp* | Integration of the *P_brpA_gfp* reporter into *ebgA* | This study |
| pMMb-tfox | Expression of TfoX | (3) |

**References**

1. **Chodur DM**, **Guo L**, **Pu M**, **Bruger E**, **Fernandez N**, **Waters C**, **Rowe-Magnus DA**. 2017. The Proline Variant of the W[F/L/M][T/S]R Cyclic Di-GMP Binding Motif Suppresses Dependence on Signal Association for Regulator Function. J Bacteriol **199**:e00344–17.

2. **Simon R**, **Priefer U**, **Puhler A**. 1983. A Broad Host Range Mobilization System for In Vivo Genetic Engineering: Transposon Mutagenesis in Gram Negative Bacteria. Nat Biotechnol **1**:784–791.

3. **Dalia TN**, **Hayes CA**, **Stolyar S**, **Marx CJ**, **McKinlay JB**, **Dalia AB**. 2017. Multiplex Genome Editing by Natural Transformation (MuGENT) for Synthetic Biology in Vibrio natriegens. ACS Synth Biol **6**:1650–1655.
