# Supplementary material for: Environmental Calcium Initiates a Feed-Forward Signaling Circuit That Regulates Biofilm Formation and Rugosity in Vibrio vulnificus
Source: mBio. 2018 Aug 28;9(4):e01377-18. doi: 10.1128/mBio.01377-18 (PMC6113621; doi:10.1128/mBio.01377-18)
Supplement: TABLE S2 [file mbo004184044st2.docx]

**Table S2. Primers used in this study**

| Name | Purpose | Sequence |
| --- | --- | --- |
| VV1_0726-3kbup-F | Replace cysD with TmR | CAGTTATTGTCGGCACTTGGTG |
| VV1_0726-3kbup-R | Replace cysD with TmR | GTCGACGGATCCCCGGAATCATAGTTATTTCCTTTTTAAACCTTTTG |
| VV1_0726-3kbdn-F | Replace cysD with TmR | GAAGCAGCTCCAGCCTACATAAGGATCTAAGGAAAGAAAATG |
| VV1_0726-3kbdn-R | Replace cysD with TmR | GTCACCAGTTCCGTGAGGATC |
| VV1_0727-3kbup-F | Replace cysN with TmR | CAGCACCAATACCCCGCCAATCAC |
| VV1_0727-3kbup-R | Replace cysN with TmR | GTCGACGGATCCCCGGAATTAATCGTTACCAAAGATTTAGG |
| VV1_0727-3kbdn-F | Replace cysN with TmR | GAAGCAGCTCCAGCCTACACATCTTTCCATCGAACTATTGATAAGC |
| VV1_0727-3kbdn-R | Replace cysN with TmR | GGTGTTCGACAGCTGCGTAAACG |
| cysC3kbup-F | Replace cysC with TmR | GTGGATGGTCTACAAGCGGAGCG |
| cysC3kbup-R | Replace cysC with TmR | GTCGACGGATCCCCGGAATCATAGAAAATCCTTTTTAAAACGGG |
| cysC3kbdn-F | Replace cysC with TmR | GAAGCAGCTCCAGCCTACATAGTACTCTTATCTGAATTTTTTG |
| cysC3kbdn-R | Replace cysC with TmR | GCCAATACCATTTCAGGCACCGATC |
| cysH3kbup-F | Replace cysH with TmR | CTTACCAAGCTGGGTGCGACGC |
| cysH3kbup-R | Replace cysH with TmR | GTCGACGGATCCCCGGAATTCAAGCATAGAAATCCCTTTTAG |
| cysH3kbdn-F | Replace cysH with TmR | GAAGCAGCTCCAGCCTACATAATAATAGAGAAGCTAAAAGGC |
| cysH3kbdn-R | Replace cysH with TmR | GATGGTCCCCCCATATTCAGACAG |
| TmR-F | Amplify TmR or KanR | ATTCCGGGGATCCGTCGAC |
| TmR-R | Amplify TmR or KanR | TGTAGGCTGGAGCTGCTTC |
| gyrA1 | qRT-PCR primer | AGATCACGCCCGTAAACATTGA |
| gyrA2 | qRT-PCR primer | TGCCTAGTACATTCATCGCGT |
| SADQPCR-3 | qRT-PCR primer | CCCATTTACCCACGGCTCAT |
| SADQPCR-4 | qRT-PCR primer | TAGACACGCTCTTTCGCTCG |
| brpJ-3kbup-F | Replace brpJ with TmR | CACCTTTAAGGAAAAATGGGCTTTG |
| brpJ-3kbup-R | Replace brpJ with TmR | GTCGACGGATCCCCGGAATCATAAAATGATTCTCAAATATATAG |
| brpJ-3kbdn-F | Replace brpJ with TmR | GAAGCAGCTCCAGCCTACATAGAGACATGAGAAAATTAGGAT |
| brpJ-3kbdn-R | Replace brpJ with TmR | GATAATGCGGTAAGTCGTTCAATC |
| brpF-3kbup-F | Replace brpF with TmR | GTTATTTCGTCACGTCCCTAAAAG |
| brpF-3kbup-R | Replace brpF with TmR | GTCGACGGATCCCCGGAATCATGATGCTTCGTTCTTTATCTCAAG |
| brpF-3kbdn-F | Replace brpF with TmR | GAAGCAGCTCCAGCCTACATGATTAACCGTCAAAATTTCAATAC |
| brpF-3kbdn-R  cysJQPCR-1  cysJQPCR-2 | Replace brpF with TmR  qRT-PCR primer  qRT-PCR primer | GAGTCGGAAAGCGTTGGTGCTTAG  TGGAACAAGAGGCCAAAGCA  GGCATTATCTGGCGCTTCAC |
