# Supplementary material for: Environmental Calcium Initiates a Feed-Forward Signaling Circuit That Regulates Biofilm Formation and Rugosity in Vibrio vulnificus
Source: mBio. 2018 Aug 28;9(4):e01377-18. doi: 10.1128/mBio.01377-18 (PMC6113621; doi:10.1128/mBio.01377-18)
Supplement: TABLE S3 [file mbo004184044st3.docx]

**Table S3. Tn insertions that disrupt calcium-induced *P_brpA_* expression.**

| Locus tag | gene |
| --- | --- |
| *aot11_00085* | *brpT* |
| *aot11_11065* | next to *yfgL* |
| *aot11_12925* | catalyzes the transfer of 2-keto-3-deoxy-D-manno-octulosonic acid to lipid A |
| *aot11_10870* | *dnaK* promoter |
| *aot11_00995* | phosphotransferase system mannitol |
| *aot11_12580* | sulfate adenylyltransferase subunit 2 CDS |
| *aot11_10475* | Mg2+/Co2+ transporter |
| *aot11_09315* | GGDEF domain-containing protein |
| *aot11_07240* | OMP |
| *aot11_19625* | glyoxylase II family protein CDS |
| *aot11_01130* | with MglBC transports galactose or methyl galactoside into the cell; contains 2 ATP binding domains |
| *aot11_08175* | H-NS promoter |
| *aot11_20010* | GGDEF domain-containing protein |
| *aot11_00235* | GGDEF domain-containing protein |
| *aot11_00025* | promoter of *brpA* |
| *aot11_11220* | ABC-type phosphate transport system, ATPase component |
| *aot11_03865* | hypothetical 81aa protein |
| *aot11_11230* | ABC-type uncharacterized transport system, permease component |
| *aot11_01125* | ABC-type sugar transport system, periplasmic component |
| *aot11_06040* | AraC-type DNA-binding domain-containing protein |
| *aot11_06030* | DNA/RNA helicase |
| *aot11_15350* | OmpC superfamily outer membrane |
