# Supplementary material for: Environmental Calcium Initiates a Feed-Forward Signaling Circuit That Regulates Biofilm Formation and Rugosity in Vibrio vulnificus
Source: mBio. 2018 Aug 28;9(4):e01377-18. doi: 10.1128/mBio.01377-18 (PMC6113621; doi:10.1128/mBio.01377-18)
Supplement: TABLE S4 [file mbo004184044st4.docx]

**Table S4. Sulfate assimilation pathway genes regulated BrpT and c-di-GMP**

|  | **Fold-change relative to WT** | |
| --- | --- | --- |
| **Gene** | **WT-v/WT-DcpA** | **∆*brpT-DcpA*/WT-DcpA** |
| *cysC* | 3 | -3.5 |
| *cysN* | 3.4 | -7.7 |
| *cysD* | 5.8 | -14.9 |
| *cysH* | -3.5 | NC |
| *cysI* | -4.7 | NC |
| *cysJ* | -3.6 | NC |
| *cysK* | -10.5 | NC |

Values show the fold-change in wildtype carrying the empty expression vector (WT-v) or the *brpT* mutant expressing DcpA (∆*brpT*-DcpA) relative to wildtype expressing DcpA (WT-DcpA). Green shading denotes up-regulation and red shading indicates down-regulation.
